# The Significance of MET Expression and Strategies of Targeting MET Treatment in Advanced Gastric Cancer

**DOI:** 10.3389/fonc.2021.719217

**Published:** 2021-09-07

**Authors:** Haiyan Liao, Tiantian Tian, Yuling Sheng, Zhi Peng, Zhongwu Li, Jingyuan Wang, Yanyan Li, Cheng Zhang, Jing Gao

**Affiliations:** ^1^National Cancer Center/National Clinical Research Center for Cancer/Cancer Hospital & Shenzhen Hospital, Chinese Academy of Medical Sciences and Peking Union Medical College, Shenzhen, China; ^2^Department of Gastrointestinal Oncology, Key Laboratory of Carcinogenesis and Translational Research (Ministry of Education/Beijing), Peking University Cancer Hospital and Institute, Beijing, China; ^3^Department of Oncology, The First Affiliated Hospital of Shandong First Medical University & Shandong Provincial Qianfoshan Hospital, Jinan, China; ^4^School of Medicine, The Southern University of Science and Technology, Shenzhen, China; ^5^Department of Pathology, Key Laboratory of Carcinogenesis and Translational Research (Ministry of Education/Beijing), Peking University Cancer Hospital and Institute, Beijing, China

**Keywords:** MET expression, advanced gastric cancer, real-time, chemotherapy, HER2

## Abstract

**Background:**

Accurate assessment of predictive biomarker expression is critical in patient selection in clinical trials or clinical practice. However, changes in biomarker expression may occur after treatment. The aim of the present study was to evaluate the effects of chemotherapy on MET expression in gastric cancer (GC).

**Methods:**

MET expression was examined immunohistochemically before and after treatment in 122 patients with unresectable or recurrent GC, and was evaluated according to H-score or the scoring criteria used in the MetMAb trial. *MET* gene amplification was assessed by chromogenic *in situ* hybridization (CISH). The antitumor effect of MET targeted therapy was investigated in human gastric cancer cells *in vitro* and *in vivo*, and the underlying molecular mechanisms were analyzed by western blot.

**Results:**

MET expression was associated with Lauren classification as well as tumor differentiation by either scoring system. MET amplification was not associated with clinical characteristics. Of the 71 patients who had paired pre- and post-treatment tumor tissues, 28 patients (39%) were initially positive for MET expression, and 43 (61%) were negative. Twenty-five patients (35%) showed significant changes in MET expression after treatment (P=0.007). Additionally, there was a concomitant overexpression of MET and HER2 in a subset of GC patients. MET inhibitor volitinib could significantly inhibit cell proliferation and xenograft growth *in vitro* and *in vivo* in MKN45 cells with MET and phosphorylated MET (pMET) high expressions *via* suppressing downstream PI3K/Akt and MAPK signaling pathways. Furthermore, combination therapy targeting both MET and HER2 demonstrated a synergistic antitumor activity.

**Conclusions:**

MET expression is altered post chemotherapy and MET status should be evaluated in real-time. Both MET and pMET expressions might need to be considered for patients suitable for volitinib treatment.

## Introduction

Gastric cancer (GC) is the third most common cancer in China, causing more than 373,000 deaths annually (1). Because of its insidious nature, patients newly diagnosed with GC often present with advanced unresectable disease. In patients with resectable cancer who undergo surgery, recurrence is common. For those patients with advanced cancer, chemotherapy is the main treatment. However, the efficacy of chemotherapy is limited, and novel targeted therapies are urgently needed.

In addition to human epidermal growth factor receptor 2 (HER2), hepatocyte growth factor receptor (MET) has recently emerged as an important target in GC. Aberrant constitutive activation of the MET signaling pathway frequently occurs in multiple neoplasms, including GC (2–6). MET overexpression with or without gene amplification resulting in the activation of MET signaling, involving MAPK, PI3K/Akt, and STAT3 signalings, is the most frequent mechanism causing GC (7–14). Moreover, higher *MET* gene amplification and expression have been associated with poor prognosis in GC (15, 16).

Several drugs have been developed to target the MET signaling axis, one of which is onartuzumab, a monovalent anti-MET antibody. Although onartuzumab activity was observed in early clinical trials, the MetGastric and the MetLung phase III studies on onartuzumab in gastric and lung cancer showed negative results, which were attributable to various reasons, including patient selection (17, 18). Another possible reason is that the tissue collected at the time of initial diagnosis may not have provided a good estimate of the level of MET expression because tumor characteristics may be altered after chemotherapy (19, 20). S. van de Ven et. al (21). reviewed 32 studies that investigated the concordance between hormone receptors and HER2 after neoadjuvant chemotherapy (NAC) in breast cancer and found a change in hormone receptors and HER2 status. To date, no study has investigated whether chemotherapy affects MET expression, which should be addressed to guide the development of innovative precision medicine and to optimize future therapies. Furthermore, recent studies have highlighted the importance of MET in trastuzumab resistance (22, 23). Previous studies suggest that co-expression of HER2 and MET influences trastuzumab resistance (24–26), which is likely due to the activation of the extensive cross-talk between MET and HER family (27), as they share key downstream MAPK or AKT signaling nodes. However, whether HER2 and MET are co-expressed has not yet been fully studied in Chinese GC patients, and whether the combination of HER2 and MET targeted therapies can synergistically inhibit tumor growth has not been well established.

The present study evaluated the effects of treatment on MET expression using pre- and post-treatment paired samples and different immunohistochemistry (IHC) scoring criteria. The expression profile of MET and HER2 as well as the prognostic role of MET were also investigated. We also tried to explore the efficacy and mechanisms of a novel MET inhibitor volitinib both in GC cell lines and PDX model.

## Materials and Methods

### Study Population and Clinical Data Collection

Pre-treatment tumor samples from patients with unresectable/recurrent GC who were enrolled between January 2008 and August 2013 were collected after obtaining patient informed consent and ethics committee approval from Peking University Cancer Hospital (PUCH). The biopsy tumor samples were obtained by gastroscopy from the primary GC site. The tissues were fixed in formalin and then paraffin-embedded in blocks, which were prepared at the time of biopsy. Medical data including age, gender, clinical diagnose, previous treatments, tumur stage according to the TNM classification system, tumor grade, the histologic classification of based on Lauren’s criteria, HER2 test results, and survival follow-up information were collected from medical records. MET protein expression and gene amplification testing of all formalin-fixed paraffin-embedded (FFPE) tumor samples, and the testing data were collected.

To compare MET protein expression and *MET* gene copy number in biopsy *vs* resection specimens, some biopsy (n=81) and resection (n=72) samples that were preserved in AstraZeneca laboratory were also detected and analyzed.

### IHC for MET Expression

Four-micrometer-thick tumor sample sections were freshly cut from the archival tumor FFPE blocks for MET IHC analysis to evaluate MET protein expression levels. IHC detection of MET was performed using a rabbit monoclonal anti-total MET antibody (SP44, Ventana Medical Systems, Inc., Roche, Tucson, AZ, USA) on an automatic immunostainer (Discovery XT, Ventana Medical Systems, Inc.). A minimum of 100 tumor cells was examined for MET staining. Staining intensity (0, negative; 1, weak; 2, moderate; 3, strong) and prevalence of these intensities in tumor cells were evaluated. The tumours were scored according to the criteria used in the onartuzumab (MetMAb) phase II trial in GC, where MET-positive samples were defined as having ≥50% of tumor cells showing moderate or strong intensity MET staining (28). Another evaluation was performed using the hybrid (H)-score, in which each individual intensity level (0-3) was multiplied by the percentage of cells with that intensity, and all values were added to obtain the final H score, which ranged from 0 to 300. MET staining patterns, i.e. membranous *vs* cytoplasmic, were assessed and recorded.

### Chromogenic *In Situ* Hybridization

Four-micrometer-thick tumor sections were freshly cut from archival tumor FFPE blocks for *MET* CISH analysis to assess *MET* gene copy number. *MET* gene copy number per cell was determined by CISH technique using both *MET*-specific and centromere 7 (*CEP7*)-specific probes following the manufacturer’s recommendations (Ventana Medical Systems, Inc.). A minimum of 50 non-overlapping cells with hybridization signals were examined for each sample. Mean *MET* gene and mean *CEP7* copy number per cell and *MET/CEP7* ratio were recorded. Tumors with *MET*/CEP7 ratios ≥ 2.0 (designated as “amplified”) and/or *MET* ≥ 4.0 copies (named “high polysomy”) were considered *MET* CISH-positive.

### MET Fluorescence *In Situ* Hybridization

Four-micrometer-thick tumor sections were freshly cut from archival tumor FFPE blocks for *MET* FISH analysis. *MET* FISH evaluation was performed on unstained FFPE tissue sections using a *MET*/CEP7 probe cocktail (Dako) according to the manufacturer’s instructions. A minimum of 50 non-overlapping cells with hybridization signals from each sample were examined under a fluorescence microscope (Olympus, BX53). Tumors with *MET*/CEP7 ratios ≥ 2.0 (named “amplified”) and/or *MET* ≥ 5.0 copies (named “high polysomy”) were considered *MET* FISH-positive (29).

### HER2 Expression Evaluation

HER2 expression was retrospectively collected based on the medical records. HER2 expression was analyzed by IHC using the PATHWAY anti-HER-2/neu (4B5) rabbit monoclonal primary antibody (Ventana Medical Systems, Inc.). In cases of IHC 2+ staining, FISH was performed. Tumor specimens with a *HER2: CEP 17* signal ratio ≥2.0 were considered *HER2* FISH-positive. As previously reported (30), HER2 positivity was defined as IHC 3+ staining alone or the combination of IHC 2+ staining and FISH-positive.

### Cell Lines and Reagents

Human gastric cancer cell lines SNU-216, MGC803, MKN45, NCI-N87 and BGC823 were purchased from the cell bank of Peking Union Medical College (Beijing, China). All cells were incubated at 37℃ in a humidified incubator with 5% CO_2_ and cultured in RPMI 1640 medium (Gibco-BRL, MD, USA) supplemented with 10% fetal bovine serum (Gibco-BRL) and 1% penicillin and streptomycin (Gibco-BRL). Volitinib was kindly provided by AstraZeneca Pharmaceuticals (Cambridge, UK). Trastuzumab (Herceptin) was purchased from Roche Pharmaceutical Ltd. (Shanghai, China). Recombinant human HGF was purchased from R&D Systems.

### Cell Viability Assay

Cells were seeded into 96-well plates at a density of 3,000~5,000 cells/well and allowed to adhere overnight. Cells were incubated with volitinib with 2-fold serial dilutions alone or the combination of trastuzumab in culture medium in the presence or absence of 20 ng/mL HGF for 72 hours. After treatment, cell proliferation was measured using a CCK8 kit (Dojindo Laboratories, Tokyo, Japan) according to the manufacturer’s protocol. Absorbance was measured at 480 nm using spectrophotometry.

### Western Blot Analysis

Cells were harvested at 70~80% confluence and were lysed using a CytoBuster protein-extraction reagent (Merck Millipore, Darmstadt, Germany), containing complete protease inhibitor and phosphatase inhibitor cocktail (Roche, Basel, Switzerland). The extracted protein concentrations were quantitated using a BCA protein-assay Kit (Beyotime, Haimen, China). After protein electrophoresis and membrane transfer, the membranes were probed with primary antibodies overnight at 4°C and incubated with the secondary antibodies for 1 hour at room temperature. The signals were detected using the Clarity Western ECL substrate (Bio-Rad Laboratories Inc., Hercules, CA, USA). The primary antibodies were as follows: anti-MET antibody (#8198), anti-pMET antibody (#5605), anti-AKT antibody (#9272), anti-pAKT antibody (#9271), anti-ERK antibody (#4695), anti-pERK antibody (#4370), anti-S6 antibody (#2217), anti-pS6 antibody (#4858) purchased from Cell Signaling Technology. Anti-β-actin antibody (Lot #014 M4759) was purchased from Sigma-Aldrich (St. Louis, MO, USA).

### *In Vivo* Animal Experiments

Two kinds of xenograft models were included in this study. Non-obese diabetic/severe combined immunodeficiency (NOD/SCID) female mice with 6-week-old (Beijing HFK Bioscience Co., Ltd., China) were inoculated with 1×107 MKN45 cells suspended in PBS to establish MKN45-derived xenograft. Three gastric cancer patient-derived xenograft (PDX) models were obtained as describes as our previously published report (31). When tumors reached about 200 mm^3^, mice were randomly assigned to vehicle control or treatment groups with at least five mice per group. Mice in treatment groups were given volitinib at a dose of 10mg/kg daily by oral gavage or trastuzumab at 20 mg/kg weekly by intraperitoneal injection, or in combination for three weeks. Tumors were measured with digital calipers twice a week, and tumor volumes were calculated by the formula: volume = length × (width)^2^/2. Tumor growth inhibition (TGI) was calculated using the following formula: TGI% = [1-(ΔT/ΔC)] × 100% (ΔT represents the tumor volume change of the drug-treated group, ΔC represents the tumor volume change of vehicle control group). All animals were performed in accordance with the guidelines approved by independent ethics committee of Peking University Cancer Hospital.

### Statistical Analysis

Statistical analysis was performed using the SPSS 18.0 software. Chi-square test or Fisher’s exact test were used to evaluate the association of clinical characteristics with MET expression and MET amplification. A Mann-Whitney test or Kruskal-Wallis test was used to analyze the relationship between MET H-scores and clinical characteristics. Overall survival (OS) was estimated and analyzed by a Kaplan-Meier curve and Cox regression model. The lo-rank test was used to detect differences in OS among various groups. For the *in vitro* and *in vivo* studies, differences between the groups were analyzed using one-way ANOVA or unpaired two-tailed *t* test. A two-sided P value < 0.05 was considered statistically significance.

## Results

### Patient Characteristics and MET Expression

**Figure 1:**
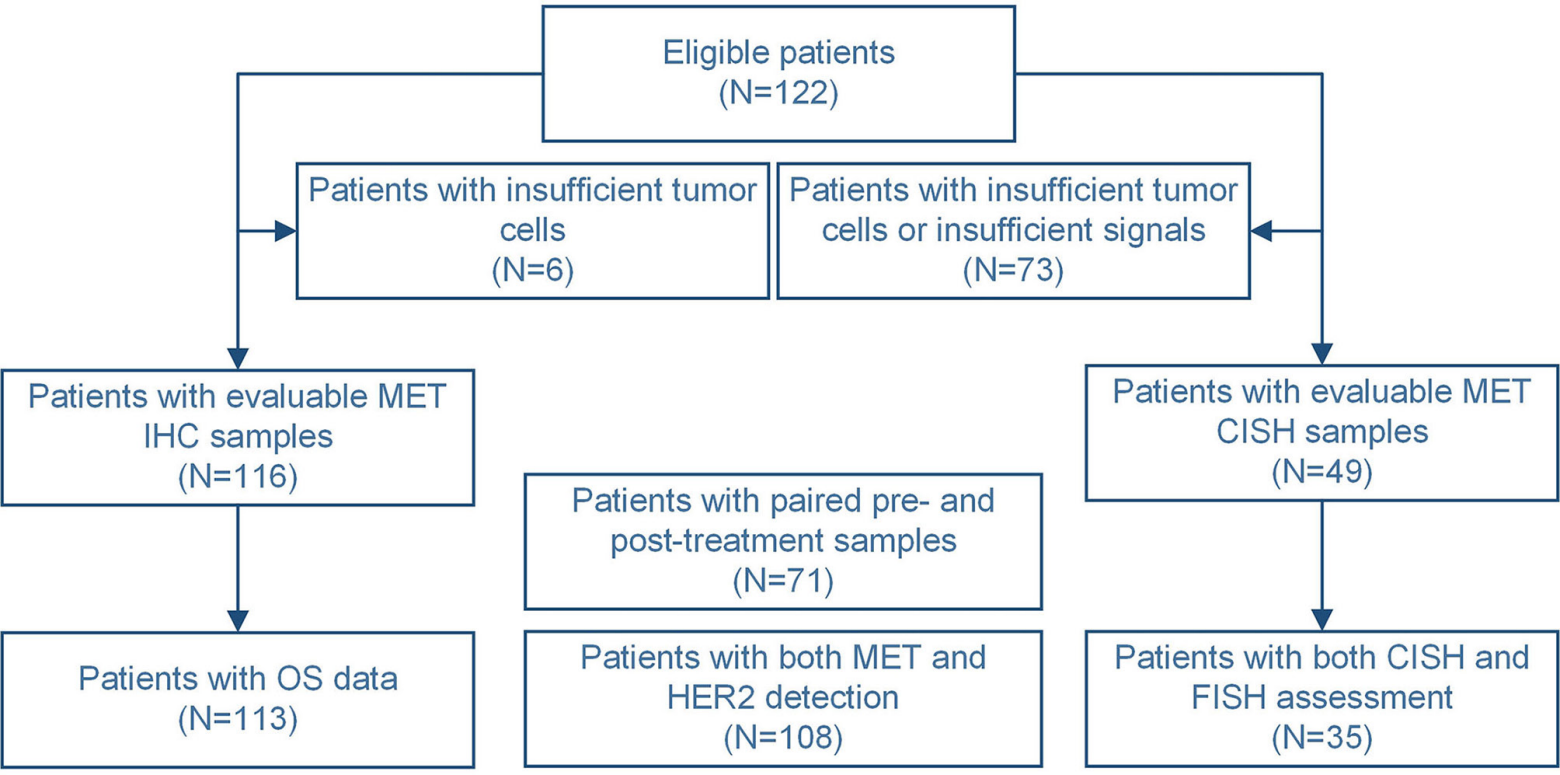
The flow diagram of patients eligible for the detection in this study.

A total of 122 patients were eligible in this study, of which tumor sections from 116 patients were assessed in terms of MET staining, but not for 6 patients due to insufficient number of tumor cells in the prepared sections. Of these 116 patients, 71 had paired pre- and post-chemotherapy samples. The schematic flow diagram of the analysis is shown in **Figure 1**. MET was localized primarily in the cytoplasm and cell membrane.

According to the MetMAb scoring criteria, 47% of pre-treatment samples (54/116) were positive. **Table 1** shows that there were no significant associations between MET protein expression and gender or age. However, Lauren classification (*P*<0.001) indicated an association between tumor grade (*P*<0.001) and tumor location (*P*=0.047) with MET expression. The rate of MET-positive cells was higher in well-differentiated and intestinal tumors, as well as tumors located in the antrum. H-score assessment indicated a median MET H-score of 130, which was chosen as the cut-off value for classifying MET-positive and MET-negative tumors. No significant differences in MET expression based on gender, age, and primary tumor site were detected. However, significant associations were observed between MET H-score and Lauren classification (*P*<0.001) and tumor grade (*P*<0.001).

**Table 1:** Patients demographics and disease characteristics by IHC.

	IHC analysis					
	All	Positive	Negative	P value	Median H-score	P value
Median age, (years)	58	59	57			
Sex				0.145		0.817
Male	92	46 (50%)	46 (50%)		130	
Female	24	8 (33%)	16 (67%)		105	
Lauren classification				<0.001		<0.001
Intestinal	60	38 (63%)	22 (37%)		160	
Diffuse	45	12 (27%)	33 (73%)		90	
Mixed	11	5 (45%)	6 (55%)		100	
Tumor differentiation				<0.001		<0.001
Well-differentiated	56	35 (63%)	21 (37%)		160	
Poorly-differentiated	60	19 (32%)	41 (68%)		90	
Tumor location				0.047		0.031
Antrum	23	16 (70%)	7 (30%)		190	
Fundus/body	40	16 (40%)	24 (60%)		105	
Gastro-esophageal junction	53	22 (42%)	31 (58%)		120	

Only 49 patients in this study were eligible for *MET* CISH analysis. The average *MET* gene copy number per cell was 2.4, and the range was from 2.0 to 11.02. Seven patients (14%) showed *MET* amplification. Of the 7 patients, 6 patients (85.7%) were MET IHC-positive, whereas 1 patient was MET IHC-negative. No significant association between *MET* amplification and gender, age, primary tumor site, Lauren classification, or tumor grade was detected, which would be further validated in future large samples.

### MET Staining in Biopsies *vs* Resections

Together with samples collected from the AstraZeneca laboratory, MET protein expression and MET gene copy number were compared in biopsy *vs* resection specimens (**Table 2**). In terms of biopsy samples, no significant differences in MET expression (40.7% *vs* 46.6%) or gene copy number (12.9% *vs* 14.3%) between the AstraZeneca laboratory and PUCH laboratory were detected. However, we observed a higher MET protein staining in biopsy specimens compared to resection ones from the AstraZeneca laboratory (20.8% *vs* 40.7%; *P*=0.008), as well as in the PUCH laboratory (20.8% *vs* 46.6%; *P*<0.001) (**Table 2**).

**Table 2:** Prevalence of MET protein expression and gene copy number based on sample types.

	AstraZeneca data		PUCH data
	Resection (FISH n=68; IHC n=72)	Biopsy (FISH n=54; IHC n=81)	Biopsy (CISH n=49; IHC n=116)
IHC positive	20.8% (n=15)	40.7% (n=33)	46.6% (n=54)
Mean *MET* gene number >4	10.3% (n=7)	12.9% (n=7)	14.3% (n=7)

### Changes in MET Expression After Chemotherapy

**Figure 2:**
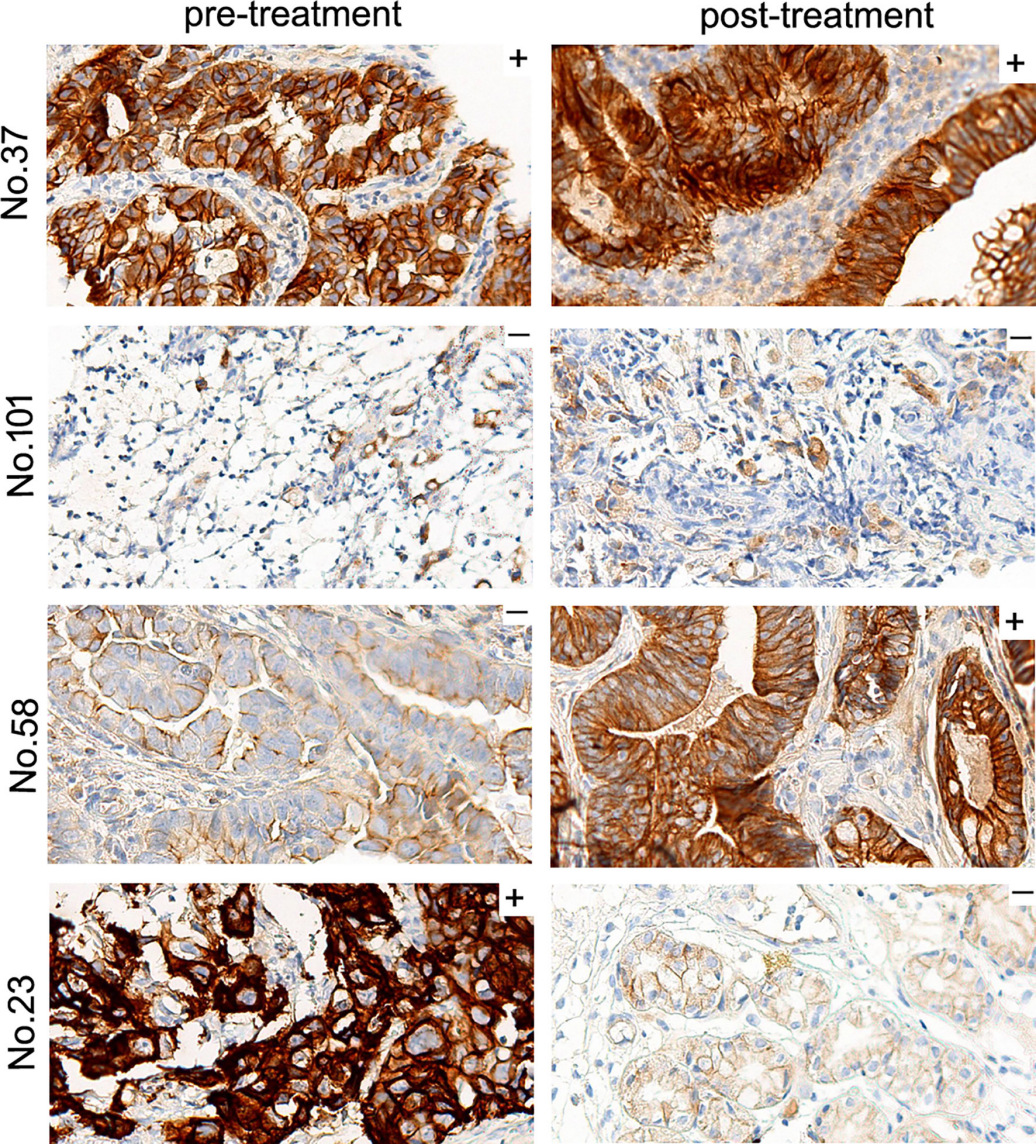
Representative IHC images of MET expression before and after treatment with chemotherapy ± trastuzumab. Based on the MetMAb scoring criteria, MET expression of patient No.37 and No.101 remained positive or negative after treatment. On the other hand, previously MET-negative No.58 exhibited positive MET staining after treatment, and formerly MET-expressing No.23 turned into MET-negative post-treatment (original magnification, 40×).

A total of 71 patients had paired pre- and post-treatment tumor tissues collected at baseline and after 6 weeks of first-line chemotherapy with fluoropyrimidine/platinum-based chemotherapy ± trastuzumab. Using the MetMAb scoring criteria, we compared the MET expression in the paired samples. Of the 71 patients, 28 patients (39%) were initially positive for MET expression and 43 (61%) were negative. There were 25 patients (35%) who showed changes in MET expression after treatment. **Table 3** and **Figure 2** show that 18 of 43 (42%) tumors with negative MET expression prior to treatment exhibited positive expression after chemotherapy, whereas 7 of 28 (25%) pre-treatment samples with positive MET expression demonstrated negative expression after treatment. We next analyzed changes in MET H-scores of the paired samples. Of the 71 paired tumors, 9 (13%) samples remained unchanged and 42 (59%) showed an increase, whereas 20 (28%) exhibited a decrease in MET H-scores after chemotherapy (data not shown). Further analysis did not detect any association between MET expression and clinical response to chemotherapy.

**Table 3:** Comparison of MET positivity by MetMab criteria of paired pre- and post-chemotherapy samples.

Pre-treatment	Post-treatment		
	+	-	All
+	21	7	28
-	18	25	43
All	39	32	71

### Association Between MET Expression and HER2 Status

In our cohort, HER2 and MET data from pre-treatment samples from 108 patients were available. Of these, 30 (28%) were HER2-positive and 78 (72%) were HER2-negative. HER2 and MET positivity were not mutually exclusive; there were 18 MET-positive patients in the 30 HER2 positive patients (60%). Notably, compared to the HER2-negative group, there were more MET-positive patients in the HER2-positive group (60% *vs* 36%, *P*=0.03) (**Table 4**).

**Table 4:** Association between MET expression and HER2 expression.

	MET-positive	MET-negative	All	*P* value
HER2-positive, n (%)	18 (60)	12 (40)	30	0.023
HER2-negative, n (%)	28 (36)	50 (64)	78	
All	46	62	108	

### Survival Analysis

We evaluated the association between pre-treatment MET expression and survival using various H-score values. Of the 116 patients whose tumor tissues were obtained prior to treatment, 113 had available survival data. The median follow-up time was 374 days. There was a numerical but not statistically significant difference in OS when the MET H-score cut-off was 130, as defined by the median H score [median OS, 319 days (95% CI: 226-412) *vs* 374 days (309-439), *P*=0.274]. However, when an H score=200 was used to define MET positivity/negativity, we observed a worse OS in the MET-positive group than in the MET-negative group [332 days (249-415) *vs* 374 days (309-439), *P*=0.023]. In the multivariate Cox regression model adjusted for other variants, a statistically significant difference in OS between the MET-positive and MET-negative patients was also observed (HR 1.6; *P*=0.025).

### Volitinib Inhibited the Growth of MKN45 Cell With MET and Pmet High Expressions *In Vitro* and *In Vivo*

**Figure 3:**
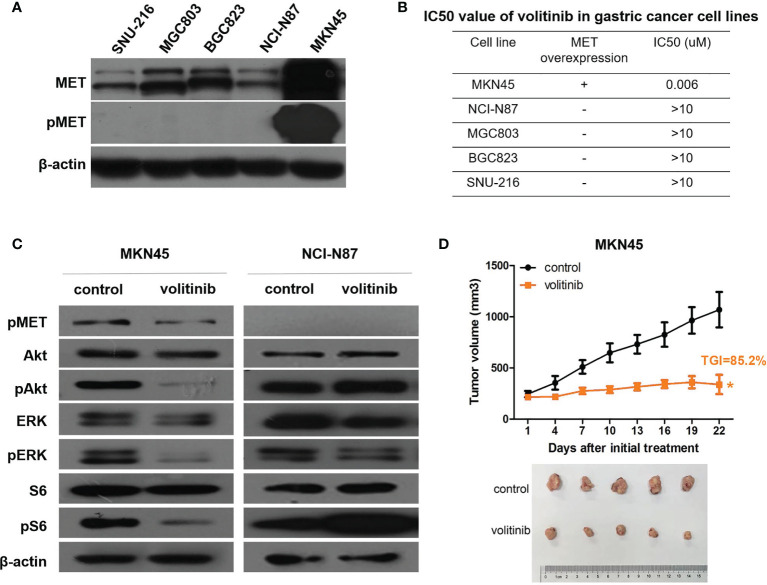
Volitinib demonstrated great antitumor activity in MET and pMET high expression MKN45 cells and xenograft. **(A)** MET and pMET expressions of 5 gastric cancer cell lines were measured by Western blotting. **(B)** The sensitivity of volitinib in 5 gastric cancer cell lines with different MET expression. **(C)** Volitinib significantly inhibited the growth of xenografts derived from MKN45 cells with TGI (tumor growth inhibition) 85.2%. Tumor volume was expressed as SD ± SEM. *P < 0.05 according to repeated measures ANOVA. **(D)** The expressions of pMET and several critical molecules involved in MAPK and PI3K/Akt pathways were monitored by Western blotting before and after volitinib treatment in MKN45 and NCI-N87 cells (NCI-N87 cell line as a control).

MKN45 cell line was featured as MET and phosphorylated MET (pMET) high expressions compared to other gastric cancer cells (**Figure 3A**), which was chosen to explore the efficacy of volitinib *in vivo* and the possible mechanisms. As expected, MKN45 cell (IC50 value = 0.006uM) was much more sensitive to volitinib than other cells (IC50 values > 10uM; **Figure 3B**), suggesting that both pMET and MET expressions might be the predictive marker of volitinib. Meanwhile, volitinib could significantly inhibit the growth of xenograft derived from MKN45 cells *in vivo* with the tumor growth inhibition (TGI) of 85.2% (*P*<0.05; **Figure 3C**). The mechanism investigation showed that volitinib played its role *via* suppressing pMET and downstream PI3K/Akt and MAPK signaling pathways indicated as decreased phosphorylated Akt, ERK, and S6 expressions (NCI-N87 cell as control; **Figure 3D**).

### Volitinib Combined With Trastuzumab Had a Synergistic Activity in Gastric Cancer PDX Models

**Figure 4:**
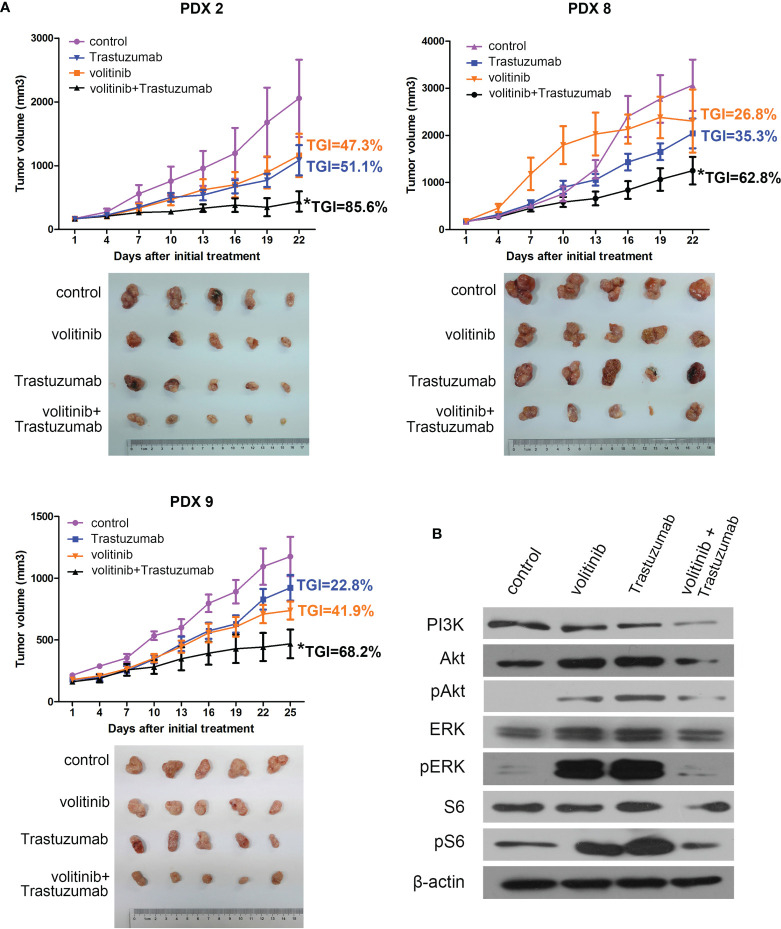
Volitinib combination with Trastuzumab showed synergistic antitumor effect in both MET and HER2 positive gastric cancer PDX models. **(A)** The *in vivo* efficacy of volitinib combination with Trastuzumab was evaluated in three both MET and HER2 positive PDX models. The combination treatment displayed significant antitumor activity with TGI 85.6%, 62.8%, 68.2% in PDX 2, PDX 8 and PDX 9, respectively, whereas either volitinib or trastuzumab alone had only weak or mild suppressive effects (TGIs 26.8%-47.3% and 22.8%-51.1%, respectively). Tumor volume was expressed as SD ± SEM. *P < 0.05 according to repeated measures ANOVA. **(B)** The expressions of several critical molecules involved in MAPK and PI3K/Akt pathways in xenograft of PDX 2 were monitored by Western blotting in control or different treatment groups. The loading samples of Western blotting were the mixture from five mice of each group of PDX 2.

Since our analysis found there was a concomitant overexpression of MET and HER2 in a subset of GC patients, the activity of volitinib combined with trastuzumab was explored in three gastric cancer PDX models with HER2 and MET positive expressions. From **Figure 4A**, both volitinib alone and trastuzumab alone exerted weak or mild tumor suppression in all PDX models with TGIs 26.8%-47.3% and 22.8%-51.1%, respectively. However, the combination therapy of volitinib and trastuzumab played a synergistic antitumor activity with TGIs 85.6% in PDX 2, 62.8% in PDX 8, and 68.2% in PDX9. For mechanism investigation, tumor tissues from PDX 2 with relative high TGI of combined therapy were analysed. Either volitinib or trastuzumab alone could induce the negative feedback rather than the inhibition of MAPK and PI3K/Akt singling pathways, suggesting the poor efficacy of single drug treatment in patients with both MET and HER2 high expressions (**Figure 4B**). Consistent with the **Figure 4A**, after the treatment of volitinib combined with trastuzumab, the downstream MAPK and PI3K/Akt singling pathways were not activated, indicating the high antitumor activity.

## Discussion

There is currently no standard definition for MET positivity in gastric cancer (32). In the present study, we explored the association between MET expression and clinical characteristics in 122 Chinese patients with gastric cancer. MET positivity was found to be associated with intestinal and moderately differentiated gastric cancer, and similar results were obtained with the two scoring systems. When the H-score of MET expression was defined as ≥200, MET-positive patients had poorer survival, which needs to be further validated in future larger cohorts.

We demonstrated that MET protein expression is altered post chemotherapy in advanced gastric cancer. In the present study, a significant increase in MET expression was observed after first-line therapy, and it is possible that tumor cells that overexpress MET may be more resistant to chemotherapy (33). Although no association between MET expression and clinical response was detected in the present study, the correlation between MET expression and drug resistance should be further investigated. Our findings emphasize the importance of a repeat testing for MET overexpression to guide MET-directed therapy after first-line treatment failure. Real-time detection of MET expression is critical in the design of MET pathway inhibitor clinical studies and accurate treatment regimens. In addition to the potential differential response of MET overexpressed tumor cells to chemotherapy, changes in MET expression may be due to tumor heterogeneity in gastric cancer (34, 35), which is currently considered as a major challenge to cancer treatment.

The addition of trastuzumab to standard chemotherapy can significantly improve the OS of patients with HER2-positive advanced gastric cancer (36). However, the response rate to trastuzumab-based treatment in this patient group is less than 50%. The existence of co-activated pathways including MET may be one of the possible reasons for resistance to HER2-targeted treatment (37). Similar to the finding of previous studies (25, 38), we demonstrated that there was a concomitant overexpression of MET and HER2 in a subset of patients with gastric cancers, suggesting the feasibility of dual blocking of MET and HER2. In our study, volitinib combined with trastuzumab had a significant synergism in three gastric cancer PDX models compared to volitinib or trastuzumab alone (**Figure 4**), which was not evident in NCI-N87 cell with HER2 high expression and MET low expression (**Supplementary Figure S1**). Our results provided a rationale for further clinical studies of targeting MET and HER2 therapies for GC patients. The concomitant expression of MET and HER2 in post-treatment samples was not assessed in this study and should be investigated in future to better understand its correlation after the first line therapy.

Both surgical specimens and biopsy samples can be used for MET testing in gastric cancer, and MET status may vary among patients. The present study observed a higher rate of MET staining in biopsy samples compared to that in resections. Cancer stage may be an important factor that contributes to MET staining as late-stage cancer is generally characterized by MET overexpression (7, 39, 40) and surgical specimens are usually obtained from early-stage patients, whereas biopsies are more commonly obtained from late-stage patients. Actually, in clinical practice, many patients could not obtain the biopsy sample after chemotherapy due to different reasons. As discussed above, real-time detection of MET expression should be carried out as far as possible in order to guide precision treatment in clinical practice. Moreover, if biopsy samples were not obtained, other methods such as liquid biopsy could be used.

CISH and FISH are two assays used in assessing gene amplification, and the consistency of results between CISH and FISH for some markers such as HER2 is very high. Compared to FISH, CISH can readily be performed using a light microscope and the staining results can be easily photographed, and slides may be stored for extended periods time (41, 42). Currently, there are no approved methods for evaluating MET gene amplification. In the present study, we compared the CISH and FISH results of 35 patients. Five cases were identified as amplified using CISH but not FISH, and only one case was determined to be amplified using either FISH or CISH (data not shown). Spearman’s correlation coefficient between the two assays was 0.25, indicating the need to validate the CISH findings on *MET* amplification, as well as establish a standard method for this particular procedure.

In summary, MET expression is altered after chemotherapy, indicating that MET status should be re-evaluated after chemotherapy in real-time. MET expression was associated with Lauren classification and tumour differentiation, and MET H-scores ≥200 suggest poor prognoses in gastric cancer patients. This study also demonstrated a concomitant overexpression of MET and HER2 in a subset of patients with gastric cancers. A combination of MET and HER2 targeted therapies exerted a significant synergism in gastric cancer PDX models, suggesting this combination strategy may be a potential therapeutic option for some gastric cancer patients and warrants a further investigation in clinical studies.

## Data Availability Statement

The raw data supporting the conclusions of this article will be made available by the authors, without undue reservation.

## Ethics Statement

The animal study was reviewed and approved by Peking University Cancer Hospital. Written informed consent was obtained from the individual(s) for the publication of any potentially identifiable images or data included in this article.

## Author Contributions

JG and CZ conceived and designed the study. HL, TT and YS were involved in study performing, the data collection, data analysis, and writing of the manuscript. ZP, JW, and YL obtained the patient’s samples and assisted in performing experiments. ZL evaluated the results of experiments. All authors contributed to the article and approved the submitted version.

## Funding

This work was supported by the National Natural Science Foundation of China (grant numbers 82072728), and Sanming Project of Medicine in Shenzhen, China (SZSM201812062).

## Conflict of Interest

The authors declare that the research was conducted in the absence of any commercial or financial relationships that could be construed as a potential conflict of interest.

The reviewer CD declared a shared affiliation with one of the authors YS to the handling editor at the time of review.

## Publisher’s Note

All claims expressed in this article are solely those of the authors and do not necessarily represent those of their affiliated organizations, or those of the publisher, the editors and the reviewers. Any product that may be evaluated in this article, or claim that may be made by its manufacturer, is not guaranteed or endorsed by the publisher.
