# Supplementary material for: The Significance of MET Expression and Strategies of Targeting MET Treatment in Advanced Gastric Cancer
Source: Front Oncol. 2021 Sep 7;11:719217. doi: 10.3389/fonc.2021.719217 (PMC8453156; doi:10.3389/fonc.2021.719217)

Supplementary Figure S1. The synergistic effect of volitinib combined with Trastuzumab in NCI-N87 cell was not evident. (A) Cell survival of NCI-N87 treated with 1 μM volitinib or/and 1 nM Trastuzumab in the present of HGF for 72 hours. No significant difference was found in combination group compared to single drug groups. (B) The expressions of several critical molecules involved in MAPK and PI3K/Akt pathways in NCI-N87 cell were monitored by Western blotting. HGF means hepatocyte growth factor. Vol and Tra represents volitinib and Trastuzumab, respectively.

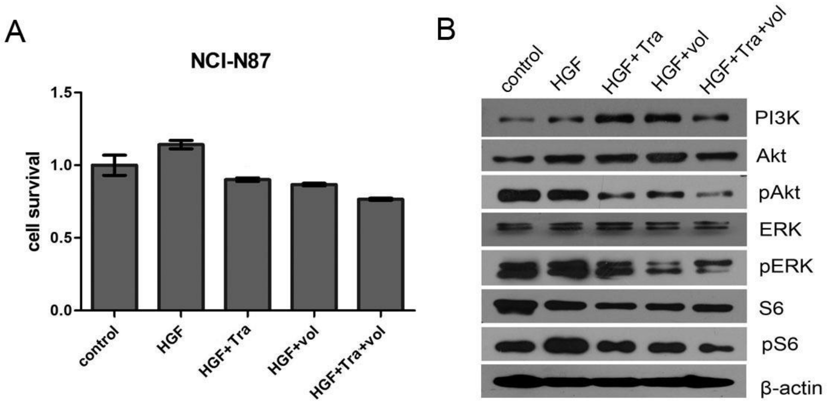

Supplement: Supplementary file 1 [file DataSheet_1.pdf]
